# Are screen-based sedentary behaviors longitudinally associated with dietary behaviors and leisure-time physical activity in the transition into adolescence?

**DOI:** 10.1186/1479-5868-10-9

**Published:** 2013-01-25

**Authors:** Mekdes K Gebremariam, Ingunn H Bergh, Lene F Andersen, Yngvar Ommundsen, Torunn H Totland, Mona Bjelland, May Grydeland, Nanna Lien

**Affiliations:** 1 Department of Nutrition, Faculty of Medicine, University of Oslo, Oslo, Norway; 2 Department of Coaching and Psychology, Norwegian School of Sport Sciences, Oslo, Norway; 3 Department of Sports Medicine, Norwegian School of Sport Sciences, Oslo, Norway

**Keywords:** Children, Adolescents, Sedentary behaviors, Screen time, Dietary behaviors, Physical activity, Associations, Longitudinal

## Abstract

**Background:**

There is a need for more longitudinal studies investigating the associations between screen-based sedentary behaviors (SB), dietary behaviors and leisure-time physical activity (PA).

**Methods:**

In the HEIA cohort study, 908 children were followed from age 11 to age 13 (September 2007 – May 2009). The children self-reported their intake of fruits, vegetables, soft drinks with sugar and snacks. TV/DVD use, computer/game use and leisure-time PA were also self-reported. Multilevel generalized linear mixed model analysis was used to assess longitudinal associations between the screen-based SB and each of the two other behaviors.

**Results:**

Twenty-month changes in TV/DVD use and computer/game use were positively associated with changes in the consumption of soft drinks with sugar and unhealthy snacks in the same period; and inversely associated with change in vegetable consumption. Change in computer/game use was also inversely related to change in fruit consumption. An inverse but non-substantive association was found between change in TV/DVD use and change in leisure-time PA. Change in computer/game use was not significantly associated with change in leisure-time PA.

**Conclusions:**

Changes in screen-based SB were associated with multiple unfavorable changes in dietary habits, although the associations were weak. These associations need to be further investigated in intervention/experimental studies, to assess whether changing screen-based SB will result in clinically relevant changes in dietary behaviors. However, the findings of this study suggest that screen-based SB and leisure-time PA are largely independent behaviors which should be addressed separately in health promotion activities.

## Introduction

Several reviews of cross-sectional, longitudinal and experimental studies indicate that SB play a role in the development of overweight/obesity in children, and that reducing SB might help reduce overweight/obesity [1-7]. Differences in this relationship across sex and type of SB investigated have been reported in some of the reviews [2-4]. The magnitude of this association has also been described as small [2]. Suggested mechanisms linking SB to overweight/obesity include the associations between SB and 1) dietary behaviors and 2) physical activity (PA) [5]. These associations can be explored cross-sectionally. However, such an approach does not allow for the exploration of temporal relationships; adopting a longitudinal approach helps address this weakness. The presence of longitudinal associations might imply that targeting one behavior might help improve or worsen the other behavior, depending on directions of association.

SB such as television (TV) viewing are believed to be associated with dietary behaviors through several mechanisms. One of these is the influence of food advertisements on food choice and intake [5,8-10]. The consumption of highly advertised foods might in turn lead to the replacement of less advertised foods such as fruits and vegetables. SB such as TV, computer or game use are also found to serve as dishabituators or distractors. This disruption of habituation to food cues leads to overeating and thereby to an increase in energy intake [11,12]. A recent systematic review highlighted the existence of consistent associations between SB and unfavorable dietary behaviors in all age groups [13]. Nonetheless, most of the studies included were cross-sectional and TV viewing was the predominantly assessed SB. A need for more longitudinal studies investigating the association between dietary behaviors and SB, and a need for inclusion of SB other than TV use were expressed [13].

The other potential mechanism relating SB with body weight is the possibility of SB displacing PA [5]. The evidence in support of this hypothesis is however conflicting. Whereas several studies have shown an inverse relationship between screen-based SB and PA [14-17], other studies, including a large meta-analysis indicated that the relationship is weak, making its practical significance limited [2,18-20]. Other studies documented no associations between SB and PA during adolescence [21-24]. Much of the evidence to date is however derived from cross-sectional studies.

The aim of the present study was to assess the longitudinal associations between SB (TV/DVD use and computer/game use) and 1) dietary behaviors (intake of soft drinks with sugar, unhealthy snacks, fruits and vegetables) and 2) leisure-time PA, over a period of 20 months among Norwegian children in the transition phase between childhood and adolescence.

## Methods

### Design and sample

The participants in the HEIA cohort study are students from 25 control schools of the HEalth In Adolescents (HEIA) intervention study [25]. A total of 177 schools were invited, and 37 schools accepted the invitation. Schools were included in this study if they had a minimum of 40 enrolled pupils in the 6^th^ grade. Schools were thus recruited from the largest towns/municipalities in seven counties from the Eastern part of Norway. Schools were identified from the website of The Norwegian Directorate for Education and Training. Twenty five of the schools were randomly assigned to the control group and twelve to the intervention group.

All 6^th^ graders in the 25 control schools included in this study and their parents/legal guardians were invited to participate in the baseline (BL) study which took place in September 2007. Parental consent was obtained for 1014 children from these schools, with a rate of parental consent of 73%. A total of 975 students (96% of the 1014 returning parental informed consent) from these schools participated at BL. A follow-up (FU) was conducted in May 2009. A total of 908 students participated at both time points. The number of participants who replied to the questions assessing SB, dietary behaviors and leisure-time PA at both time points is shown in Table 1. Ethical clearance was obtained from the Regional Committees for Medical Research and the Norwegian Social Science Data Service.

**Table 1:** Dietary intakes, TV/DVD and computer/ game use and leisure-time physical activity among Norwegian children

		**BL**		**T2**		**Change**	
	**n**	**Mean**	**CI**	**Mean**	**CI**	**Mean**	**CI**
Fruits (times/wk)	896	9.81	9.36, 10.26	9.55	9.10, 9.99	−0.26	−0.74, 0.22
Vegetables (times/wk)	881	11.05	10.42, 11.69	10.50	9.92, 11.09	−0.55	−1.24, 0.15
Soft drinks with sugar(dl/wk)	861	5.29	4.90, 5.69	6.12	5.68, 6.56	0.83	0.40, 1.26
Snacks (times/wk)	870	3.11	2.90, 3.31	3.50	3.28, 3.71	0.39	0.14, 0.64
TV/DVD use (hrs/wk)	889	11.55	11.10, 11.99	13.41	12.94, 13.88	1.86	1.38, 2.34
Computer/game use (hrs/wk)	882	8.64	8.22, 9.06	10.61	10.14, 11.09	1.97	1.48, 2.47
Leisure-time PA (hrs/wk)	877	5.97	5.72, 6.23	6.91	6.66, 7.15	0.93	0.67, 1.19

Snacks include candies/chocolate and salty snacks.

### Data collection

The children answered an internet-based questionnaire over a period of approximately 45 minutes. The children were taken to separate computer rooms in their schools in groups. Research assistants were present to answer questions, resolve technical problems and ensure that children replied independently from each other.

### Measures

#### Sedentary behaviors

Four questions with pre-coded answer categories were asked to assess usual TV/DVD use and usual use of computer/electronic games: How many hours do you usually watch TV and/or DVD on a normal weekday? The same question was asked for a normal weekend day. The answer categories were (recoding in brackets): half hour or less [0.5], one hour [1], two hours [2], three hours [3], four hours [4], five hours or more [5]. The two questions on computer/ electronic game use were formulated in the same way as for TV/DVD, but the answer categories were: no playing [0], half hour or less [0.5], one hour [1], two hours [2], three hours [3], four hours or more [4]. Weekly scores for TV/DVD and computer/electronic games were calculated by summing hours reported for an average weekday (multiplied by five) and average weekend day (multiplied by 2).

Adequate test-retest reliability for these SB measures has previously been documented [26].

#### Dietary behaviors

Consumption of fruits and vegetables (raw and cooked) was assessed using the following questions: “How often do you usually eat fresh fruit?”, “How often do you usually eat raw vegetables?” and “How often do you usually eat cooked vegetables?” There were eight response categories ranging from never/seldom to three times per day or more. The questions assessing intake of fruits and vegetables were validated among 11-year-olds with a 7-day food record as the reference method, and were found to have a satisfactory ability to rank subjects according to their intake of fruits and vegetables [27].

Frequency of consumption of snacks (sweet snacks (candies/chocolate) and salty snacks (potato chips, popcorn etc.)) was assessed using two questions with seven response categories ranging from never/seldom to two times per day or more; frequencies of consumption of sweet snacks and salty snacks were added to create a total snack intake variable.

Intake of soft drinks with sugar during weekdays was assessed using a frequency question (with response categories ranging from never/seldom to every weekday); and a question assessing amount in glass (with response categories ranging from one to four or more). The children were informed that ½ liter = 3 glasses; one glass was then converted to 1.67 deciliters. Frequency and amount were multiplied to get a weekday consumption measure. The children were also asked about the amount of soft drink with sugar consumed in the weekends using questions with response categories ranging from never/seldom to 7 glasses or more. When a range was provided, mid-points were used while recoding. Weekday and weekend values were summed up to create a weekly intake variable. The questions assessing the intake of soft drinks with sugar have been validated among 9- and 13-year-olds using a 4-day precoded food diary as the reference method, and moderate Spearman correlation coefficients were obtained [28]. However, separate assessment of weekday and weekend intake was not made in the validation study.

Adequate test-retest reliability for all the measures of dietary behavior has also previously been documented [29].

#### Leisure-time physical activity

Leisure-time PA was assessed using the questions: “On weekdays, after you have left school, how often do you work out, play, and engage in sports/physical activity (e.g. ball games, skiing, dance, and gymnastics, playing outside)?” The answer categories were: Never or almost never, 1–2 times per week, 3–4 times per week, 5–6 times per week, 7–8 times per week, 9–10 times per week. Duration was assessed using the question: “When you work out, play, engage in sports/physical activity after you have gone from school, how often are you active every time (on average)?” The answer categories were: less than 20 minutes, 20–40 minutes, 41minutes-1hour, more than 1 hour. Similar questions were asked for weekends. “During weekends, how often do you work out, play, engage in sports/physical activity (includes activities in sports clubs, youth club, scouts, walks in the woods)”. The answer categories were: almost never or never, 1 time per weekend, 2 times per weekend, 3–4 times per weekend, 5–6 times per weekend. Duration was assessed in a similar manner as for weekdays. Recoding was done using mid-points when a range was provided. Frequency and duration were multiplied to obtain a measure of total leisure-time PA. Weekday and weekend values were summed up to create a weekly leisure-time PA variable.

The questions used to assess leisure-time PA were based and modified from a previously validated self-reported questionnaire [30]. A test-retest reliability coefficient of 0.69 was obtained for the weekly leisure-time PA measure.

Due to their empirical and theoretical associations with the different health behaviors explored, gender, parental education and weight status were adjusted for in all analyses.

Parental education was gathered as part of the parental informed consent for the adolescent. It was categorized into: low (12 years or less), medium (between 13 and 16 years) and high (more than 16 years). Educational status of the parent with the longest education or else the one available was used in the analyses.

Body mass index (BMI) was calculated as weight/height^2^. Weight and height were objectively measured by same sex project staff. The age and gender specific BMI cut-off values proposed by the International Obesity Task Force were used in order to categorize the adolescents into non-overweight and overweight/obese [31].

### Statistical analysis

Mean values were calculated for the outcome measures at BL and FU. Change between these time points was also computed, by subtracting FU values from BL values (FU-BL). In order to assess the longitudinal associations between SB, dietary behaviors and leisure-time PA, multilevel generalized linear mixed model analysis, which accounts for the clustering of students within schools, was used.

Adjusted associations of change in TV/DVD use and change in computer/electronic game use with change in dietary behaviors were estimated in a set of regression models. Changes in dietary behaviors were used as outcome variables in these sets of regression analyses; change in TV/DVD use and change in computer/game use were used as predictors. Baseline TV/DVD use and baseline computer/game use were also included in the respective models. Adjustment for gender, BL weight status, parental education and BL values of the dietary behaviors was made. Adjustment for these BL values allows for the correction of the phenomenon of regression to the mean [32], as BL values were found to be significantly associated with FU values.

Adjusted associations of change in the SB with change in leisure-time PA were assessed using a set of regression analyses with change in leisure-time PA as outcome and change in TV/DVD use and computer/game use as predictors. Baseline TV/DVD use and baseline computer/game use were also included in the respective models. Adjustment for gender, parental education and weight status, as well as for BL leisure-time PA was done.

In addition, to account for correlations between change in TV/DVD use and change in computer/game use, change in TV/DVD use was included as a covariate in models where change in computer/game use was used as predictor and vice versa.

All statistical analyses were performed by IBM® SPSS® Statistics, version 19.0 (IBM Corp., Somers, New York, USA).

## Results

The mean age at BL was 11.2 y (SD = 0.3, range = 10.5−12.5). Of the participants included, 48% were females. The proportion of children with parents with high education was 33% and that of children with parents with medium education was 36%. At BL, 14.5% of the children were found to be overweight/obese (data not shown).

The mean values at both time points and changes between the time points in the SB, dietary behaviors and leisure-time PA are presented in Table 1.

### Longitudinal associations between SB and dietary behaviors

Small but significant inverse associations between change in TV/DVD use and change in vegetable consumption over 20 months were found in the regression analyses. The association between change in TV/DVD use and change in fruit consumption was also inverse but non-significant. Similarly, small but significant positive associations were found between change in TV/DVD use and change in soft drink with sugar consumption and change in snacks’ consumption over 20-months (Table 2). Inverse associations between change in computer/game use and change in fruit and change in vegetable consumption, and positive associations between change in computer/game use and change in soft drink with sugar consumption and change in snacks’ consumption over 20-months were also found (Table 3).

**Table 2:** Longitudinal associations between TV/DVD use and dietary behaviors over 20 months among Norwegian children

	**Change in fruit consumption (times/wk)**		**Change in vegetable consumption (times/wk)**		**Change in soft drinks with sugar consumption (dl/wk)**		**Change in snack consumption (times/wk)**	
	**Est**	**C.I**	**Est**	**C.I**	**Est**	**C.I**	**Est**	**C.I**
1	−0.05	−0.12, 0.01	−0.13	−0.22, −0.03	0.23	0.16, 0.29	0.12	0.09, 0.15
2	−0.12	−0.19, −0.05	−0.19	−0.29, −0.10	0.13	0.06, 0.20	0.07	0.03, 0.10

N varies between 808 and 831.

Est= estimate, C.I. = confidence interval.

Multilevel generalized linear mixed model analysis, the estimates indicate the change in the dietary behaviors associated with: 1. an hour per week increase in TV/DVD use over 20 months.

2. an hour increase in television viewing at baseline.

All changes are weekly changes.

Adjustment for BL consumption of the respective food items, gender, weight status, parental education and change in computer/game use was done.

**Table 3:** Longitudinal associations between computer/game use and dietary behaviors over 20 months among Norwegian children

	**Change in fruit consumption (times/wk)**		**Change in vegetable consumption (times/wk)**		**Change in soft drinks with sugar consumption (dl/wk)**		**Change in snack consumption (times/wk)**	
	**Est**	**C.I**	**Est**	**C.I**	**Est**	**C.I**	**Est**	**C.I**
1	−0.13	−0.19, −0.06	−0.12	−0.21, −0.03	0.14	0.08, 0.20	0.06	0.03, 0.10
2	−0.08	−0.16, −0.01	−0.15	−0.26, −0.05	0.10	0.05, 0.20	0.06	0.03, 0.11

N varies between 801 and 835.

Est= estimate, C.I. = confidence interval.

Multilevel generalized linear mixed model analysis, the estimates indicate the change in the dietary behaviors associated with: 1. an hour per week increase in computer/game use over 20 months.

2. an hour increase in computer/game use at baseline.

All changes are weekly changes.

Adjustment for BL consumption of the respective food items, gender, weight status, parental education and change in TV/DVD use was done.

In addition, BL TV/DVD use was found to be independently inversely associated with 20-month changes in the consumption of fruits and vegetables; and to be independently positively associated with changes in the consumption of soft drinks with sugar and snacks. Similarly, computer/game use at age 11 was independently inversely associated with changes in the consumption of fruits and vegetables; and was independently positively associated with changes in the consumption of soft drinks with sugar and snacks over 20 months (Table 3). Adjustment for PA did not significantly change the associations between SB and the dietary behaviors and therefore associations unadjusted for PA are presented.

All significant associations between screen-based SB and dietary behaviors were significant at the 0.01 level, except for the association between BL computer/game use and change in fruit consumption, which was significant at the 0.05 level.

### Longitudinal associations between SB and leisure-time PA

Change in TV/DVD use was found to be inversely associated with change in leisure-time PA over 20 months (Table 4). This association, although statistically significant (p=0.03), was however not substantive, with an hour per week increase in TV/DVD use over 20 months being associated with a 0.04 hour per week decrease in leisure-time PA.

**Table 4:** Longitudinal associations between leisure-time physical activity (PA) and sedentary behaviors over 20 months among Norwegian children

**Change in leisure time PA related to 1-hr increase in TV/DVD use**		**Change in leisure time PA related to 1- hr increase in baseline TV/DVD use**		**Change in leisure time PA related to 1-hr increase in computer/game use**		**Change in leisure time PA related to 1-hr increase in baseline computer/game use**	
**Est**	**C.I**	**Est**	**C.I**	**Est**	**C.I**	**Est**	**C.I**
−0.04	−0.08, −0.01	−0.05	−0.09, −0.01	−0.02	−0.06, 0.01	−0.06	−0.10, −0.02

N varies between 816 and 823.

Est= estimate, C.I. = confidence interval.

Multilevel generalized linear mixed model analysis.

All changes are weekly changes in hrs/wk.

Adjustment for baseline leisure-time PA, baseline TV/DVD use or computer/game use, gender, weight status, parental education and change in the SB not used as predictor was done.

BL TV/DVD use was also independently inversely significantly associated (p=0.02) with change in leisure-time PA but this association was also not substantive.

No statistically significant association was found between change in computer/game use and change in leisure-time PA over 20 months (Table 4).

A separate analysis of weekday and weekend measures was conducted as time available for SB and leisure-time PA is different during weekdays and weekend days, and yielded similar results (data not shown).

## Discussion

The study investigated longitudinal associations between SB and 1) dietary behaviors and 2) leisure-time PA over a period of 20 months. Findings indicated that changes in TV/DVD use and computer/game use during this period were associated with unhealthy changes in dietary behaviors; BL TV/DVD use and computer/game use were also independently associated with undesirable changes in dietary behaviors. The association between TV/DVD use and leisure-time PA was inverse, but not substantive. No significant association was found between change in computer/game use and change in leisure-time PA.

The associations between SB and dietary behaviors found in this study are in line with those of a recent systematic review of mainly cross-sectional studies [13], including a European cross-national cross-sectional study with representative samples from different countries including Norway which found TV viewing to be positively associated with the consumption of sweets and soft drinks and inversely related to the consumption of vegetables [33]. The few longitudinal studies included in the review also indicated an inverse relationship between SB and the intake vegetables [18,34]; and a positive association between TV viewing and the consumption of food items such as snacks and sugar sweetened beverages [35], which is in line with the findings of this study. This study adds to the existing literature by extending these findings to the use of computer/games. Inverse associations between TV use and consumption of fruits were also documented in the aforementioned studies. However, in the present study, this association was non-significant, which might be related, among other things, to the fact that the change in fruit consumption over 20 months was very small, making it difficult to find associations; baseline TV/DVD use was however inversely related to change in the consumption of fruits.

Although the associations between SB and dietary behaviors documented in this study are small, they have likely been attenuated by measurement errors; and a consistent pattern of unfavorable associations with several dietary behaviors investigated was found. In addition, SB at age 11 was also independently associated with the change in the dietary behaviors investigated over 20 months. The specific mechanisms behind the association between SB and dietary behaviors cannot be identified from this study, and only hypotheses can be put forward. One possibility is the exposure to advertisements which influences children’s consumption patterns [8-10], including through a higher responsiveness to food advertising among children with higher media exposure [8]. There is a ban on advertisements aimed at children during children’s TV programs in Norway. However, children’s programs broadcasted from outside of Norway do contain advertisements; the children might also watch adult-targeted TV programs containing food adverts. The children might also be exposed to advertisements on the internet. The other possibility is that parents provide some types of foods that they themselves associate with screen time to their children, starting from a young age. According to the habit theory, once habits are formed, automatic triggering of subsequent behavior occurs upon exposure to environmental cues which normally precede the action [36]. Hence, if screen-based SB have been repeatedly accompanied by consumption of unhealthy food items, these SB might become automatic cues to such dietary habits [36-38]. In a study among adolescents in the Netherlands, Kremers et al. found that more time spent on TV viewing is associated with a stronger habit for soft drink intake, and found that a quarter of the variance in habit strength of soft drink consumption could be explained by habit strength of TV use [37]. de Bruijn and van den Putte similarly found that a strong habit towards TV viewing was positively related with soft drink consumption [38]. Provision of healthy foods during screen time by parents might help disrupt such automatic activation of unhealthy eating habits. The current findings imply that a decrease in SB might result in favorable changes in dietary behaviors, although these changes might be small.

The study lends support to the findings of several studies, mainly cross-sectional, pointing to a lack of significant association between SB and leisure-time PA [21-24]. The findings are however not supportive of those of other longitudinal and cross-sectional studies indicating inverse associations between PA and SB [14-17]. The weak longitudinal association between TV/DVD use and leisure-time PA might however indicate a possibility for some displacement of PA by TV/DVD use, although this association is unlikely to be clinically relevant. Measurement errors have however likely attenuated this association. Previous studies have similarly found TV viewing to be more strongly or more consistently inversely associated with PA compared to other SB such as game use and computer use [16,17]. As stated by Marshal et al., the total amount of time per day engaged in different SB is inevitably prohibitive of PA [2]; however it is possible that not all types of PA are related to SB. In a moderator analysis in their review paper, Marshal et al. found that only vigorous PA was inversely related to TV viewing. This finding could be real, but it could also be related to the fact that vigorous PA is more easily recalled than moderate PA, making it easier to find associations [2].

It has been suggested that the link between SB and overweight/obesity is more likely to be due to associations with dietary behaviors than to associations with PA [13]. The results of this study support this suggestion.

### Limitations and strengths of the study

An important methodological issue to be taken into consideration when interpreting the results of this study is the regression dilution bias, which is a statistical phenomenon whereby random measurement errors in independent variables attenuate regression coefficients [39]. As random measurement errors in the independent variables used in this study, i.e. TV/DVD use and computer/game use exist, the weak associations found are most probably higher than documented. Such measurement errors can also shift results to the null [39].

The changes in dietary behaviors and leisure-time PA over 20 months documented in this study were rather small, which might make it difficult to find significant associations [32]. Information about amount of consumption was not available except for soft drink with sugar consumption, therefore only frequency measures were used, and adjustment for total energy intake was not possible. Finally, although temporal relationships could be assessed in this study, its observational nature does not allow for causal inferences to be made. A randomized controlled trial that alters the SB would make it possible to get a strong evidence of causality. In addition, although there is no reason to believe that a change in dietary behaviors would lead to a change in SB, it is possible that a change in PA leads to a change in SB and not vice versa. Finally, the study is restricted to a single geographical area, making generalizability limited.

The study has several strengths. Most evidence about the associations investigated in this study has to date been derived from cross-sectional studies. However, demonstrating that a change in an independent variable predicts a change in a dependent variable provides a stronger evidence of causality compared to using an independent variable measured at one point in time [40]. Adjustment for characteristics of children empirically and theoretically associated with the behaviors investigated was another strength of this study. SB other than TV use, as well as several dietary behaviors were also included. The sample size was relatively large, and the rate of retention in the study was high.

## Conclusion

Changes in screen-based SB were associated with multiple unfavorable changes in dietary behaviors, although the associations were weak. These associations need to be further investigated in intervention/experimental studies, to assess whether changing screen-based SB will result in clinically relevant changes in dietary behaviors. However, the findings of this study suggest that SB and leisure-time PA are largely independent behaviors which should be addressed separately in health promotion activities.

## Abbreviations

BL: Baseline; PA: Physical activity; BMI: Body mass index; SB: Sedentary behaviors; FU: Follow-up; TV: Television; HEIA: HEalth In Adolescents.

## Competing interests

The authors declare they have no competing interests.

## Authors’ contributions

MKG conducted the statistical analyses and wrote the first draft of the manuscript and made the greatest contribution to the paper. NL, IHB, LFA, YO, MB, MG participated in designing the study, project planning and data collection. All authors have critically revised the manuscript, and read and approved the final version of the manuscript.
